# The use of a genomic relationship matrix for breed assignment of cattle breeds: comparison and combination with a machine learning method

**DOI:** 10.1093/jas/skad172

**Published:** 2023-05-23

**Authors:** Hélène Wilmot, Tobias Niehoff, Hélène Soyeurt, Nicolas Gengler, Mario P L Calus

**Affiliations:** National Fund for Scientific Research (F.R.S.-FNRS), B-1000 Brussels, Belgium; TERRA Teaching and Research Centre, Gembloux Agro-Bio Tech, University of Liège, B-5030 Gembloux, Belgium; Animal Breeding and Genomics, Wageningen University and Research, 6700AH Wageningen, the Netherlands; TERRA Teaching and Research Centre, Gembloux Agro-Bio Tech, University of Liège, B-5030 Gembloux, Belgium; TERRA Teaching and Research Centre, Gembloux Agro-Bio Tech, University of Liège, B-5030 Gembloux, Belgium; Animal Breeding and Genomics, Wageningen University and Research, 6700AH Wageningen, the Netherlands

**Keywords:** breed assignment, genomic relationship matrix, local breeds, machine learning, single nucleotide polymorphism, support vector machine

## Abstract

To develop a breed assignment model, three main steps are generally followed: 1) The selection of breed informative single nucleotide polymorphism (SNP); 2) The training of a model, based on a reference population, that allows to classify animals to their breed of origin; and 3) The validation of the developed model on external animals i.e., that were not used in previous steps. However, there is no consensus in the literature about which methodology to follow for the first step, nor about the number of SNP to be selected. This can raise many questions when developing the model and lead to the use of sophisticated methodologies for selecting SNP (e.g., with iterative algorithms, partitions of SNP, or combination of several methods). Therefore, it may be of interest to avoid the first step by the use of all the available SNP. For this purpose, we propose the use of a genomic relationship matrix (GRM), combined or not with a machine learning method, for breed assignment. We compared it with a previously developed model based on selected informative SNP. Four methodologies were investigated: 1) The PLS_NSC methodology: selection of SNP based on a partial least square-discriminant analysis (PLS-DA) and breed assignment by classification based on the nearest shrunken centroids (NSC) method; 2) Breed assignment based on the highest mean relatedness of an animal to the reference populations of each breed (referred to mean_GRM); 3) Breed assignment based on the highest SD of the relatedness of an animal to the reference populations of each breed (referred to SD_GRM) and 4) The GRM_SVM methodology: the use of means and SD of the relatedness defined in mean_GRM and SD_GRM methodologies combined with the linear support vector machine (SVM), a machine learning method used for classification. Regarding mean global accuracies, results showed that the use of mean_GRM or GRM_SVM was not significantly different (Bonferroni corrected *P* > 0.0083) than the model based on a reduced SNP panel (PLS_NSC). Moreover, the mean_GRM and GRM_SVM methodology were more efficient than PLS_NSC as it was faster to compute. Therefore, it is possible to bypass the selection of SNP and, by the use of a GRM, to develop an efficient breed assignment model. In routine, we recommend the use of GRM_SVM over mean_GRM as it gave a slightly increased global accuracy, which can help endangered breeds to be maintained. The script to execute the different methodologies can be accessed on: https://github.com/hwilmot675/Breed_assignment.

## Teaser

The genomic relationship matrix can reliably be used, potentially combined with a machine learning method, to assign animals to their breed. With this methodology, the step of selection of a single nucleotide polymorphism panel can be avoided.

## Introduction

Developing a suitable model for breed assignment is often necessary for the management of livestock, e.g., because the pedigree is missing (especially in endangered breeds; e.g. Wilmot et al., 2022a) or because of breed-derived products traceability purposes (e.g., Judge et al., 2017; Wilmot et al., 2022b). In general, three main steps have to be followed to develop a breed assignment model: 1) Selection of breed-informative single nucleotide polymorphism (SNP), 2) Training of a classification model, and 3) Validation of the tuned classification model on external animals, i.e., that were not used for the training steps. The reasons for selecting a breed-informative SNP panel instead of all the SNP provided by the SNP chip (or at the overlap of several SNP chips) can be summarized as followed: 1) It increases the global accuracy of the model, i.e., its ability to correctly assign animals to their breed of origin (Wilkinson et al., 2011; Pasupa et al., 2020; Wilmot et al., 2022a), 2) The number of SNP highly exceeds the number of samples leading to a risk of overfitting (Pasupa et al., 2020), 3) It reduces the time needed for computation (Kwak and Choi, 2002) and 4) It can decrease genotyping costs. Even if genotyping costs are constantly decreasing, it is common to genotype animals at a minimum density (e.g., 10k or 50k SNP) and, if necessary to impute them to a higher density (VanRaden et al., 2011).

However, how to implement the first step is a complex issue most of the time. The first question to answer is which methodology should be used. In the literature, very different methodologies have been applied to select the most breed informative SNP, and there is no consensus on a universally best method. Examples include the use of fixation index (*F*_*ST*_), absolute allele frequency differences or principal component analysis (PCA; Wilkinson et al., 2011; Hulsegge et al., 2013; Judge et al., 2017; Bertolini et al., 2018). Recently, some studies have even combined several methodologies to select breed-informative SNP, which added another level of complexity. For example, Hulsegge et al. (2019) used a PCA in combination with a random forest to select SNP and Pasupa et al. (2020) used a sophisticated methodology combining information gain, a genetic algorithm and frequency feature selection for this purpose. This kind of complex methodology can also involve iterative algorithms (Pasupa et al., 2020; Moradi et al., 2021), which increases computation time to train the model. Another issue with this first step is to estimate the optimal number of SNP to allow breed classification. Again, there is no consensus in the literature about the protocol to follow. Various approaches have been used, such as: log-likelihood ratio of probabilities to be assigned to a breed (Hulsegge et al., 2013), threshold of the needed global accuracy (Wilkinson et al., 2011), or threshold of the used measure of informativeness (Wilmot et al., 2022a). During the process of SNP selection, there is also the risk to select SNP that are in linkage disequilibrium (Kumar et al., 2019), resulting in collinearity of the variables used, which may affect the performance of the classification model. Finally, another important issue is that the selection of a SNP panel implies it is specific to the studied breeds (Judge et al., 2017; Kumar et al., 2019), which means that a new SNP panel would have to be selected for a new breed to be assigned. Given these issues in the selection of the most breed-informative SNP, it may be desirable to skip this step and use all the available SNP for breed assignment.

To solve this issue, we proposed the use of a genomic relationship matrix (GRM) for breed assignment. To our knowledge, GRM have never been used directly for breed assignment. However, it has already been used indirectly for this purpose, e.g., through genomic best linear unbiased prediction (Dodds et al., 2014). Because the GRM is very widely used, e.g., for computation of genomic predictions, genetic variance within population and genetic correlations between populations, it would be interesting to extend its current use to breed assignment. The objective of this study was therefore to compare the performances of a breed assignment model based on a GRM, combined or not with a machine learning method, to a previously developed model based on machine learning techniques.

## Materials and Methods

The SNP data for the animals included in this study were previously obtained from samples collected by breeder associations based on relevant authorization by the different local authorities. Genotypes of Meuse-Rhine-Yssel (MRY) were provided by the Centre of Genetic Resources (Wageningen, the Netherlands). Genotypes of the East Belgian Red and White (EBRW) breed were provided by the Walloon Breeders Association (Ciney, Belgium) while those of the Red-Pied of the Ösling (RPO) breed were provided by the Administration of Technical Agricultural Services (Luxembourg, Grand Duchy of Luxembourg). More details about the breeding management of these two latter breeds can be found in Wilmot et al. (2022a).

### Dataset

The genotypes of three different red-pied cattle breeds were used in this study: those of the EBRW (*N* = 226), the RPO (*N* = 132), and the MRY (*N* = 292). All the animals sampled were recorded in the Herd Book of their respective breed. The three studied breeds are part of a genomic continuum as described in previous studies (e.g. Wilmot et al. 2022a, 2023) and can be considered as sister breeds, rooting from the same breed group. They are also very close geographically as the EBRW is Belgian, the RPO is Luxembourgish and the MRY is Dutch. Table 1 shows, for each breed, the number of samples and the distribution of the chips used for genotyping. Five different SNP chips were used: the BovineSNP50 Beadchip v2 and 3, the BovineHD Beadchip v12 and the EuroG MD v9-SI and v2 (Illumina, San Diego, CA, USA). The mapped SNP that are included on each of the five chips were used in the current study. The same quality control as in Wilmot et al. (2023) was followed and led to a total of 39,967 SNP.

**Table 1.** Number of samples per breed (in total, for each of the reference sets and for the validation set) and distribution of samples per chip

Breed	*N*	*N* for RS1	*N* for RS2	*N* for the validation set	Chip				
					BovineSNP50 Beadchip v2	BovineSNP50 Beadchip v3	BovineHD Beadchip v12	EuroG MD v9-SI	EuroG MD v2
East Belgian Red and White	226	113	50	113	90	65	0	34	37
Meuse-Rhine-Yssel	292	146	50	146	120	149	23	0	0
Red-Pied of Ösling	132	66	50	66	0	107	0	10	15

### Breed assignment methodologies

Four methodologies were used to predict the breed of origin. The available samples were divided in a reference and a validation set, and each of the four methodologies was used to predict the breed of origin of the samples in the validation set. The validation set was formed by the random selection of half of the available samples for each breed. Two modalities were tested for the reference set: the first one used the remaining half of the samples (hereafter referred to RS1) and the second one used 50 randomly selected animals per breed among the remaining half (hereafter referred to RS2). The objective of testing two reference sets was to determine the effect of the size of the reference population and of the balance of sample sizes across breeds on the performances of the different methodologies. The random selection of the validation set and the two modalities of the reference set were repeated 200 times in order to compare the performances of the different methodologies. The size of the different validation and reference sets are given in Table 1.

#### The PLS_NSC methodology.

It followed the methodology of the second best breed assignment model detected by Wilmot et al. (2022a), using less SNP but with a similar performance than the best model. For this methodology, the genotypes were standardized i.e., they were centered by the SNP mean and divided by the SNP SD. The reference set was first used to select the best SNP with a partial least squares-discriminant analysis (PLS-DA). For this purpose, only SNP with a major genotypic frequency lower than 0.95 in the reference set were kept. It allows to get rid of (almost) monomorphic SNP as it is first necessary to eliminate variables that are (almost) constant for the PLS-DA to work. To optimize the PLS-DA, a number of components ranging from 1 to 50 was tested in a 10-fold cross-validation (10-CV) within the reference set with the trainControl function of the caret v.6.0-93 R package (Kuhn, 2008). As the PLS-DA built a model for each of the three breeds of interest, the mean of absolute values of coefficients of SNP plus three times their SD were used as a threshold for selecting SNP. If the absolute value of a SNP coefficient was higher than this threshold for at least one of the three breeds, it was included in the SNP panel used for classification. Then, the method of the nearest shrunken centroid (NSC) was trained (Tibshirani et al., 2002), based on this SNP panel, to assign each animal of the reference set to its breed of origin. The NSC was also optimized in a 10-CV by the use of the trainControl function (caret v.6.0-93 R package, Kuhn, 2008) and the following values of the shrinkage level (delta) were tested: 0.01, 0.05, 0.10, 0.25, 0.50, 1. Once the model was built and optimized by the selected SNP panel and the adequate delta, the validation set was used to determine its performance. To assign animals to their breed of origin, the criteria of the highest probability was used. The different optimization parameters of the first methodology (number of SNP with major genotypic frequency lower than 0.95, number of components, number of SNP selected by the PLS-DA and delta), for each reference set and repetition, are available in Supplementary Table S1.

#### The mean_GRM methodology

This methodology was based on the use of a GRM. The GRM was built with the calc_grm program (Calus and Vandenplas, 2016), which can be accessed through the MiXBLUP software (ten Napel et al., 2021). Computation of the GRM involves first calculating allele frequencies (AF). However, for the to-be-assigned animal it is not possible to use the AF of the breed of the animal in question since its breed is not yet known. We therefore chose to use average AF across breeds. In the case of RS1, the reference set is imbalanced and computing average AF across all the genotype data may bias the GRM, because the AF used would be dominated by the breed with most samples. Therefore, genotypes of the reference set were used to compute the AF for each of the three breeds separately. These AF were then averaged across the breeds and the average AF were used for centring the genotypes and scaling the resulting GRM, following the first method of VanRaden (2008). It was also important to use only animals of the reference set for computing AF as classification results for validation animals should only be dependent of the composition of the reference set (and not of validation animals themselves). For each animal of the validation set, the mean relationship with the reference set of each breed was computed. Animals were assigned to the breed with which they had the highest mean relationship. The rationale behind the mean_GRM methodology was simple: on average, genomic relationships of an animal to members of its own breed are expected to be higher than to members of other breeds.

#### The SD_GRM methodology

The third methodology was a variation of the second methodology and used the SD of the genomic relationships, as computed for the mean_GRM methodology, to each breed of the reference set instead of the mean relationships. Animals were assigned to the breed with which they had the highest SD of the relationships. The rationale behind the SD_GRM methodology was that there is more variability of relationships within a breed than between them. For example, if we consider the close relatives of an animal (e.g. its parents, grandparents, siblings) that belong to the same breed and are more related to it than distant cousins that already belong to another breed, there is more variability of the relationships within the close family than to distant relatives. The relationship of one animal to distant relatives would be rather similar and therefore the variation of the relationships would be close to 0 in this case.

#### The GRM_SVM methodolgy

This methodology was a combination of genomic relationships, again as computed for mean_GRM, and a support vector machine (SVM) with a linear kernel. In SVM, other kernels can be used as well (e.g. radial, polynomial, etc). However, these other kernels are less intuitive to optimize as the number of parameters to be tuned increases. We wanted our tool to be available for use by the vast majority of scientists so we chose the linear kernel.

For the GRM_SVM, the mean and SD of the genomic relationships of animals of the reference set to animals of each breed of the reference set were computed. In this computation, self-relationships were excluded. Thus, six variables were computed: a mean and SD of the relatedness of the animal to each of the three breeds. These six variables were standardized (i.e., the mean of each variable was subtracted and they were divided by their SD) and used as an input for training a linear SVM that was optimized, as for PLS_NSC, by the use of a 10-CV with the help of the caret v.6.0-93 R package (Kuhn, 2008). The following values of cost (*C*) were tested: 0.001, 0.01, 0.1, 0.2, 0.3, 0.4, 0.5, 0.6, 0.7, 0.8, and 0.9. Supplementary Table S2 shows the optimal selected values for this parameter, based on RS1 and RS2, respectively. The mean and SD of the relationships of validation animals to the reference set, previously computed for mean_GRM and SD_GRM, were used to validate the tuned linear SVM. As implemented in the caret v.6.0-93 R package (Kuhn, 2008), the SVM scores, based on the distances to the decision boundary, are rescaled through a logistic transformation (Platt’s scaling), which allow to estimate probabilities (Lin et al., 2007). Therefore, validation animals were assigned to the breed for which they had the highest probability.

To allow comparison of the breed assignments, for each combination of reference set and methodology, the global accuracy, sensitivity and specificity for each breed were computed and averaged for the 200 repetitions. The global accuracy is defined as the percentage of correct assignment for all the validation animals while the sensitivity is defined for each breed as the percentage of correct assignment for this breed. In contrast, the specificity is defined as the proportion of animals not belonging to a specific breed that are not assigned to this breed. The SD, the minimum and maximum of global accuracies, sensitivities and specificities were also computed. With the objective to evaluate significance of differences of global accuracies, sensitivities and specificities between the followed methodologies, an adapted paired Student’s *T*-test for cases with resampling was realized within each modality of the reference set (RS1 or RS2) for each pair of methodology, following the formula of Bouckaert and Frank (2004):


$$\begin{array}{l} t=\frac{\frac{1}{n}\overset{n}{\underset{j=1}{\sum }}x_{j}}{\sqrt{\left(\frac{1}{n}+\frac{n_{2}}{n_{1}}\right){\hat{\sigma }}^{2}}} \end{array}$$

with *n* the number of repetitions, *x* the difference of global accuracies, sensitivities or specificities between two methodologies, *n*_*1*_ the number of samples in calibration (total number for global accuracy, number of one breed for sensitivities and number of the two other breeds for specificities), *n*_*2*_ the number of samples in validation (total number for global accuracy, number of one breed for sensitivities and number of the two other breeds for specificities), ${\hat{\sigma }}^{2}$ the estimated variance of the differences and *t* the observed *t* value. As there were six pairwise comparisons, differences were considered significant when *P* < 0.0083, very significant when *P* < 0.0017, highly significant when *P* < 0.00017, and extremely significant when *P* < 0.000017, using a Bonferroni correction. Following the adapted formula of the paired Student’s *T*-test, there can be a lack of power of detection in the case of RS2 because of an increase of the variance. That is why a bootstrap confidence interval of 95% (percentiles *P*_2.5_ and *P*_97.5_) was also computed for global accuracy, sensitivities and specificities of each methodology.

Finally, computation time was determined to evaluate which methodology was the most efficient. Computations were performed on the High Performance Computer (Anunna) of Wageningen University and Research using operating system Linux 4.15 Ubuntu 20.4. The processors used were Intel Xeon Gold 6130 CPU 64 bits with a base frequency of 2.10 GHz. For the computation of mean_GRM, SD_GRM, and GRM_SVM, 4 Gb of RAM were assigned while, for PLS_NSC, 16 Gb were assigned. For each methodology, one core was assigned. All the methodologies were implemented in R v.4.1.2 (R Core Team, 2021) and Rstudio 2023.03.0 + 386 (R Studio Team, 2023), except for the GRM computation that was done with the calc_grm program (Calus and Vandenplas, 2016). Supplementary File S1 is an R script detailing the mean_GRM, SD_GRM, and GRM_SVM methodologies and can be accessed as well on GitHub: https://github.com/hwilmot675/Breed_assignment.

## Results

**Figure 1.**
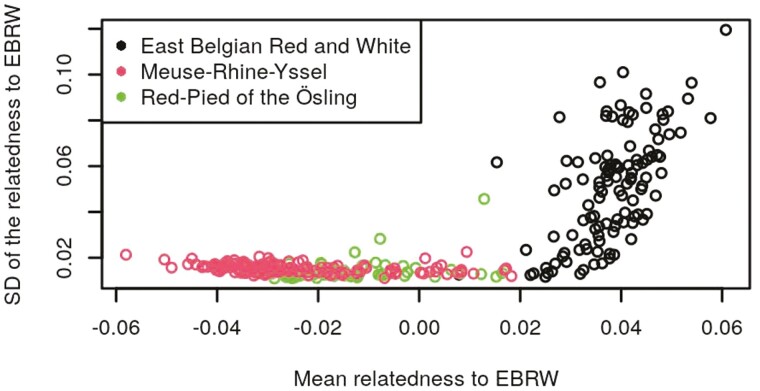
Scatterplots of the SD of the relatedness against the mean relatedness of validation animals to the EBRW breed for one repetition of reference set 1. Each dot represents a sampled animal from the validation set. Different colors represent different breeds.

**Figure 2.**
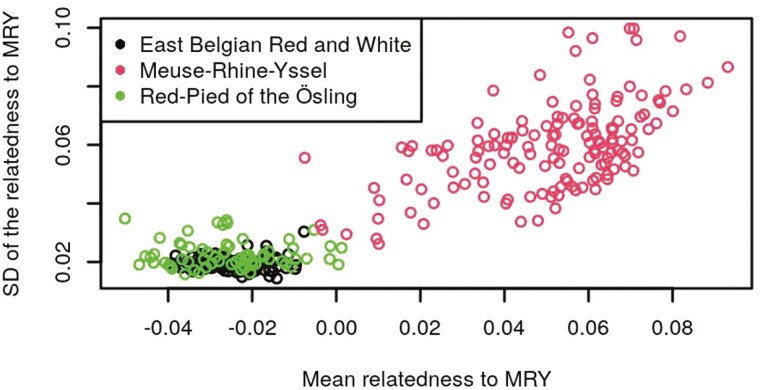
Scatterplots of the SD of the relatedness against the mean relatedness of validation animals to the MRY breed for one repetition of reference set 1. Each dot represents a sampled animal from the validation set. Different colors represent different breeds.

**Figure 3.**
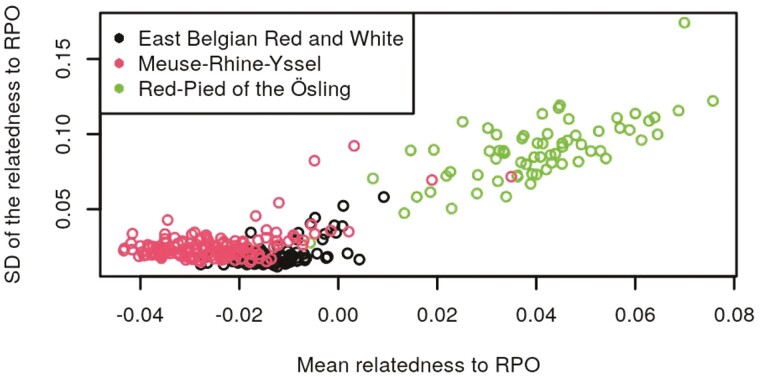
Scatterplots of the SD of the relatedness against the mean relatedness of validation animals to the RPO breed for one repetition of reference set 1. Each dot represents a sampled animal from the validation set. Different colors represent different breeds.

Figures 1 and 3 show scatterplots of the SD against the mean relatedness of validation animals with the EBRW, MRY, and RPO reference animals, for one random repetition of RS1. Supplementary Figures S1 and S3 show similar scatterplots of the SD against the mean relatedness of reference animals of each breed, within the same random repetition of RS1. As expected, it can be seen that the mean and SD of the relatedness of validation animals to their own breed were higher than those of validation animals from other breeds. This pattern was particularly obvious in Figures 1 and 2. For each breed, the correlations, averaged across repetitions, between the mean and SD of the relatedness of validation animals to the EBRW, MRY, and RPO animals of RS1, are shown in Table 2. Similar computations were made within animals of RS1 (Supplementary Table S3). Mean correlations between the mean relatedness and the SD of the relatedness ranged from 0.55 to 0.80 within breeds and from −0.19 to 0.54 across breeds. Both these figures and tables are indicating that mean and SD of the relationship were related, but included a considerable extent of different information as well.

**Table 2.** Correlations between the mean relatedness and the SD of the relatedness of each breed of the validation set to each breed of the reference set, averaged across 200 repetitions of RS1

Breed of the reference set	Breed of the validation set	Mean correlation between mean relatedness and the SD of the relatedness	SD of the correlation between mean relatedness and the SD of relatedness
EBRW	EBRW	0.63	0.083
	MRY	−0.14	0.094
	RPO	0.43	0.108
MRY	EBRW	−0.19	0.104
	MRY	0.55	0.072
	RPO	0.32	0.159
RPO	EBRW	0.50	0.083
	MRY	0.54	0.085
	RPO	0.80	0.048

**Figure 4.**
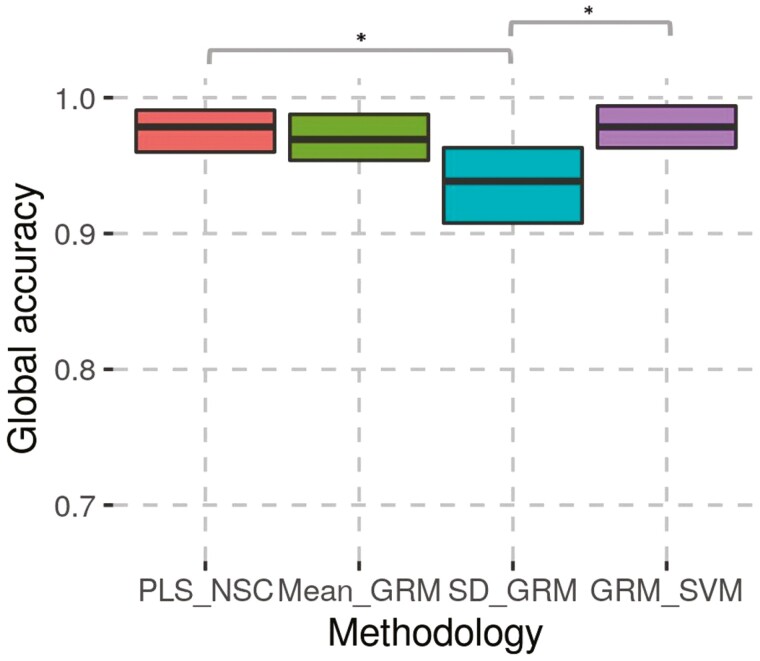
Bootstrap confidence interval of 95% and results of the pairwise Student’s *T*-test for the global accuracy for RS1. Non significant differences are not represented. *: significant difference.

**Figure 5.**
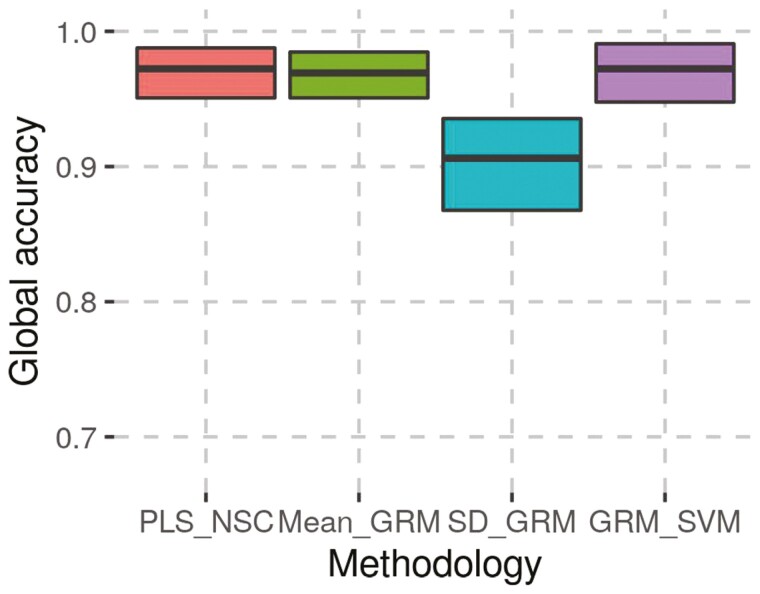
Bootstrap confidence interval of 95% and results of the pairwise Student’s T-test for the global accuracy for RS2. Non significant differences are not represented. *: significant difference.

Global accuracies for each combination of methodology and reference set, averaged across repetitions are shown in Table 3. Supplementary Table S4 shows global accuracies for each combination of methodology, reference set, and repetition. It can be observed on Table 3 that the highest mean global accuracies were obtained with GRM_SVM for RS1 and RS2. Moreover, the Student’s *T*-test did not detect any significant difference of mean global accuracies between PLS_NSC, mean_GRM, and GRM_SVM neither for RS1 (Figure 4) nor for RS2 (Figure 5). For both reference sets, the SD_GRM methodology had the lowest mean global accuracy, which showed significant differences from PLS_NSC and GRM_SVM for RS1. For the SD_GRM, the SD of the global accuracy (Table 3) and the confidence interval ­(Figures 4 and 5) were the highest. Even if, for both reference sets, the mean global accuracy of mean_GRM was not significantly different from PLS_NSC and GRM_SVM, it can be seen in Table 3 and Figures 4 and 5, that the mean and median of global accuracy were slightly lower than for PLS_NSC and GRM_SVM.

**Table 3.** Minimum, mean, maximum, and SD of the global accuracy for each combination of methodology and reference set, across 200 repetitions

Reference set	Methodology^1^			
	PLS_NSC	Mean_GRM	SD_GRM	GRM_SVM
RS1				
Minimum global accuracy, %	95.38	95.08	90.15	96.00
Mean global accuracy, %	97.86^a^	96.89^a,b^	93.82^b^	97.97^a^
Maximum global accuracy, %	99.69	99.08	97.23	99.69
SD of the global accuracy, %	0.799	0.867	1.406	0.798
RS2				
Minimum global accuracy, %	93.85	94.15	85.54	93.23
Mean global accuracy, %	97.05^a^	96.79^a^	90.38^a^	97.06^a^
Maximum global accuracy, %	99.08	99.08	95.08	99.38
SD of the global accuracy, %	0.935	0.923	1.871	1.107

^1^Methodologies with the same letter have not significantly different mean global accuracies within a reference set (*P* < 0.0083 with the Bonferroni correction).

Supplementary Tables S5 to S10 show results for the sensitivities and specificities of EBRW, MRY, and RPO, averaged across repetitions for each combination of methodology and reference set. Supplementary Table S4 shows these results for each combination of methodology, reference set, and repetition. Supplementary Figures S4 to S15 show results of the Student’s *T*-test used to detect significant differences in mean sensitivities and specificities of each breed, for each reference set. Most of the time, for both reference sets, there were no significant differences of mean sensitivities or specificities between the different methodologies. However, for RS1, a very significant difference of sensitivity of EBRW as well as a significant difference of specificity of MRY were observed between the SD_GRM and all other methodologies. For RS2, a significant difference of sensitivity of EBRW was shown between SD_GRM and the three other methodologies. However, non significant differences obtained with RS2 should be interpreted cautiously as the estimated variance of the observed *t* value increased in the used formula compared to the RS1 modality, which decreases the power of detection of the test. In Table 3, it can also be seen that all models had higher mean global accuracies when the reference set was larger. For the third methodology, the increase of mean global accuracy related to the increase of the size of the reference set was the highest (higher than 3%) while it was the lowest for mean_GRM (0.10%).

Finally, average computation times for each combination of methodology and reference set are presented in Table 4, while computation time for each combination of methodology, reference set, and repetition are presented in Supplementary Table S11. For PLS_NSC and GRM_SVM, it can be observed that computations to train the model always took more time than computations involved in predicting the breed of origin of the validation set. Moreover, PLS_NSC, with the selection of SNP, took the longest time to be trained (around 48 min on average for RS1 and around 30 min on average for RS2) but also to predict the breed of origin for new animals (a bit less than 15s for both reference sets). For all methodologies based on GRM, the total amount of time was always lower than 15s, which is very fast. Within each methodology, the total time was lower for RS2, with less animals in the reference set but the same number of animals to validate than RS1. For mean_GRM and SD_GRM, this decrease in total time was related to the decrease in time related to the computation of the GRM while, for PLS_NSC and GRM_SVM, it was related to the decrease in training time.

**Table 4.** Average computation time for each combination of methodology and reference set

Methodology	Reference set	Part of the computation	Average computation time, s
PLS_NSC	RS1	Training	2891.44
		Validation	14.97
		Total	2906.41
	RS2	Training	1786.95
		Validation	16.41
		Total	1803.36
Mean_GRM^1^	RS1	Total	6.68
	RS2	Total	4.19
SD_GRM^1^	RS1	Total	6.68
	RS2	Total	4.20
GRM_SVM	RS1	Training	11.11
		Validation	0.03
		Total	11.14
	RS2	Training	8.10
		Validation	0.03
		Total	8.13

^1^For this methodology, there is no training of the model.

## Discussion

The objective of this study was to determine the performances of the use of a GRM, combined or not with a machine learning method, for breed assignment purposes. One of the properties of the GRM, if computed using AF across all individuals included in the GRM, is that the average of all relationships is expected to be equal to 0. Therefore, the fact that we obtained negative relationships, does not really have a meaning per se (other than comparing their level to those of the other relationships), but rather is a consequence of how they were computed. Means and SD of the relatedness contained different information as shown in Figures 1 to Figure 3, Supplementary Figures S1 to S3, Table 2 and Supplementary Table S3. Especially, mean correlations within breeds were higher than across breeds and SD of the correlations were lower within breeds than across breeds, as expected. The higher mean within breed correlation obtained for RPO (0.80) can likely be explained by the smaller population size, i.e., RPO animals were probably more related to each other than EBRW/MRY animals. The mean across-breed correlations of the RPO breed were higher than other mean across-breed correlations. This is probably due to the higher mean relationships of RPO animals to EBRW and MRY breeds than those between EBRW and MRY animals. The higher variability of across-breed relationships of RPO to EBRW and MRY breeds compared to the variability of relationships between EBRW and MRY is also likely to explain the higher mean across-breed correlations. Because the mean and SD of the relatedness included different information, the idea was therefore to combine them in a single model by the use of a linear SVM (GRM_SVM). This methodology resulted in equivalent global accuracies than the use of PLS_NSC. Moreover, no significant difference was found between PLS_NSC and GRM_SVM regarding mean sensitivities and mean specificities.

When the breed assignment methodology was based on SD_GRM, the mean global accuracy was significantly lower than for PLS_NSC and GRM_SVM for RS1. Even if the difference of global accuracies between SD_GRM and other methodologies was not significant for RS2, which can be explained by the lower power of detection of the adapted Student’s *T*-test in this case, mean of global accuracies obtained was still poorer for SD_GRM than other methodologies (Table 3, Figure 5). Moreover, the decrease in mean global accuracy from RS1 to RS2 was the highest with SD_GRM. This can be partly explained by the fact that, as the reference set was smaller for RS2, validation animals had a lower probability to be closely related to one of the animals of their breed in the reference set, which decreases the SD of the relatedness to the breed they actually belong to. Moreover, the SD of the global accuracy was the highest for RS2 of SD_GRM, which means that, with a smaller reference set, this methodology was very sensitive to the animals included in the reference set. For the other methodologies, the decrease of mean global accuracy and the increase of SD from RS1 to RS2 was more marginal. Other studies have already demonstrated that the samples included in the reference set should represent their breed well to make a correct breed assignment (Funkhouser et al., 2017; Gobena et al., 2018; He et al., 2018; Hulsegge et al., 2019; Wilmot et al., 2022a). It means that not only the number of samples is important but also the representation of the variability of the population within the reference set. One follow-up of the current study might be to test the SD_GRM methodology with more samples to determine if it has similar performances than other methodologies. Unfortunately, due to limited sample sizes, it was not possible in this study.

The mean global accuracy of mean_GRM was not significantly different than those of other methodologies. However, as shown in Table 3, Figures 4 and 5, the mean and median of global accuracy were a bit lower than those of PLS_NSC and GRM_SVM. Therefore, in routine, considering the global accuracy performances, the PLS_NSC and GRM_SVM methodologies should be preferred to mean_GRM. It is particularly important in the case of endangered breeds like EBRW and RPO as animals actually belonging to the breed should be correctly detected for the maintenance of the breed and its integrity.

The main advantage of using methodologies based on GRM (mean_GRM, SD_GRM, and GRM_SVM) was to bypass the step of selection of SNP that can raise many questions about which methodology to use or how many SNP to select. Another drawback of the selection of SNP is that it is specific to the studied breeds (Judge et al., 2017; Kumar et al., 2019) and a new SNP panel would have to be selected if another breed is included. One reason advocated for a reduced SNP panel is the cost of genotyping, especially for local breeds. To reduce these costs, a SNP chip could be designed based on the selected SNP panel (Kumar et al., 2019). However, the design of a specific SNP chip is also expensive and animals are not only genotyped for breed assignment but also for other purposes as genomic diversity analysis, genomic predictions, or parentage verification. Moreover, the gap of genotyping costs between a 50k chip and a lower density chip is nowadays relatively small.

Another argument to use a reduced SNP panel is that reducing the number of features for classification problems would reduce computation time (Kwak and Choi, 2002). In our study, the total computation time was substantially lower with mean_GRM, SD_GRM, and GRM_SVM that all used all SNP, compared to PLS_NSC that used a reduced SNP panel. Comparing only the training or only the validation computation time showed that GRM_SVM was more efficient than PLS_NSC for both reference sets. A reduced validation computation time is more important in practice than a reduced training computation time because animals are assigned to their breeds in routine while training the model is performed once in a while. The problem of overfitting due to the use of a high number of SNP (Pasupa et al., 2020) was also overcome by the fact that the information found in the GRM is summarized in three variables for mean_SVM and SD_SVM and to six variables for GRM_SVM. Compared to PLS_NSC and GRM_SVM, the mean_GRM and SD_GRM had the advantage to not need any training, which allowed a relatively straightforward and therefore efficient breed assignment.

The GRM used in the different methodologies was scaled by AF of the reference set averaged across breeds. This ensures that the estimated relationships, both within the reference set and between the reference set and the new animal to be assigned, and thus the resulting breed assignment of a particular validation animal, will not be affected by the addition of other validation animals to the GRM. The scaling based on the reference set is also very practical as animals to be assigned to a breed do not have to be added and removed one by one from the GRM, but can all be included at once simultaneously with the reference animals. Thus, computation of a single GRM is sufficient for the breed assignment of all validation animals, which is not necessarily the case for other methods. For instance, Varga et al. (2022) defined a “central animal” that was the most related to the other animals of the reference population, based on an identity by state similarity matrix. However, the definition of this “central animal” was not stable as they allowed it to change with the addition of validation animals or with new animals to be assigned to a breed. Similarly, when using distances of the genotype of the animal to be assigned based on a PCA (Varga et al., 2022), the authors encountered the same problem as they computed again the principal components and the coordinates of animals when they wanted to assign a new animal to its breed. This means that the breed assignment of an animal in routine applications may be affected by the composition of the set of animals to be assigned and not only of the animals found in the reference set. This could be easily overcome by the definition of a “central animal” based only on the reference population or the projection of a new animal on the already computed components of the PCA.

## Conclusions

In this study, we demonstrated the use of a GRM-based methodology for accurate breed assignment. The methodologies based on the highest mean relationship, as defined by a GRM, and the GRM combined with a linear SVM gave similar global accuracies than the methodology based on a reduced SNP panel and the NSC method. In practice, the methodology based on a GRM combined with a linear SVM should be preferred over the one based on the highest mean relationship because it gave a slightly better percentage of correct assignment, which can be crucial for the survival of endangered breeds. The benefit of using the methodology based on a GRM and a linear SVM for breed assignment went beyond a high global accuracy; it bypassed the step of selection of SNP and required far less computation time than the NSC model based on a reduced SNP panel.

## Supplementary Material

skad172_suppl_Supplementary_Figures_S1-S15

skad172_suppl_Supplementary_File

skad172_suppl_Supplementary_Table_S1

skad172_suppl_Supplementary_Table_S2

skad172_suppl_Supplementary_Table_S3

skad172_suppl_Supplementary_Table_S4

skad172_suppl_Supplementary_Table_S5-S10

skad172_suppl_Supplementary_Table_S11

## Abbreviations:

10-CV: 10-fold cross-validation
AF: allele frequencies
*C*: cost of the linear support vector machine
EBRW: East Belgian Red and White
*F*_*ST*_: fixation index
GRM: genomic relationship matrix
MRY: Meuse-Rhine-Yssel
NSC: nearest shrunken centroids
PCA: principal component analysis
PLS-DA: partial least squares-discriminant analysis
RPO: Red-Pied of the Ösling
RS1: reference set 1
RS2: reference set 2
SNP: single nucleotide polymorphism
SVM: support vector machine

## Notes

Conflict of Interest Statement
