# Supplementary material for: The use of a genomic relationship matrix for breed assignment of cattle breeds: comparison and combination with a machine learning method
Source: J Anim Sci. 2023 May 23;101:skad172. doi: 10.1093/jas/skad172 (PMC10276639; doi:10.1093/jas/skad172)
Supplement: skad172_suppl_Supplementary_Figures_S1-S15 [file skad172_suppl_supplementary_figures_s1-s15.docx]

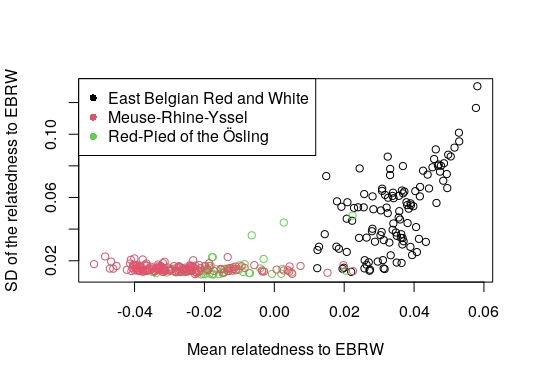


**Supplementary Figure S1.** Scatterplot of the SD of the relatedness against the mean relatedness to EBRW, within the one repetition of reference set 1. Each dot represents a sampled animal from the reference set. Different colours represent different breeds.


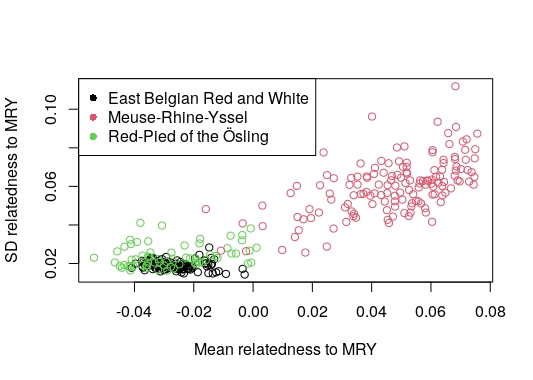


**Supplementary Figure S2.** Scatterplot of the SD of the relatedness against the mean relatedness to MRY, within one repetition of reference set 1. Each dot represents a sampled animal from the reference set. Different colours represent different breeds.


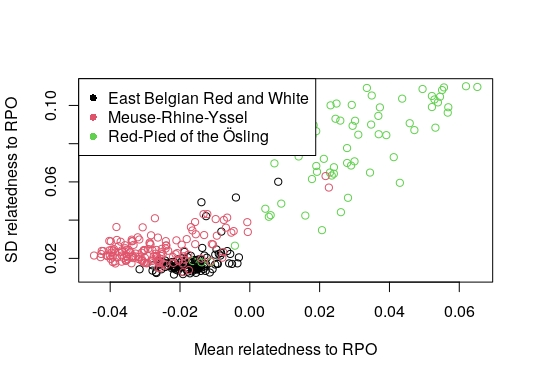


**Supplementary Figure S3.** Scatterplot of the SD of the relatedness against the mean relatedness to RPO, within one repetition of reference set 1. Each dot represents a sampled animal from the reference set. Different colours represent different breeds.


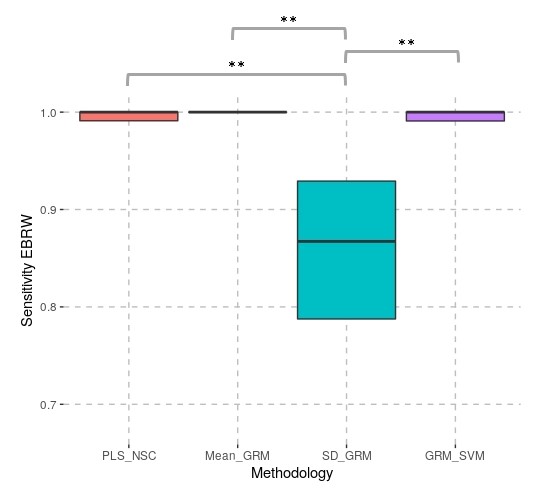


**Supplementary Figure S4.** Bootstrap confidence interval of 95% and results of the pairwise Student’s T-test for the sensitivity of EBRW for RS1. Non significant differences are not represented. **: very significant difference.


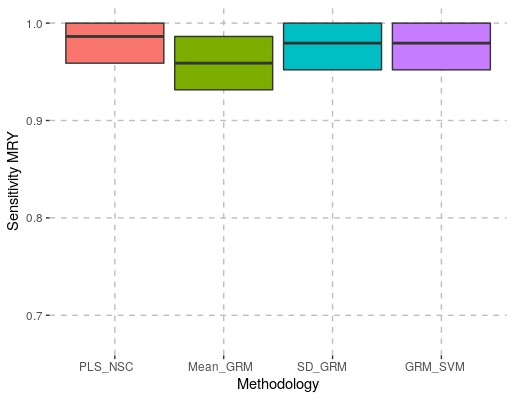


**Supplementary Figure S5.** Bootstrap confidence interval of 95% and results of the pairwise Student’s T-test for the sensitivity of MRY for RS1. Non significant differences are not represented.


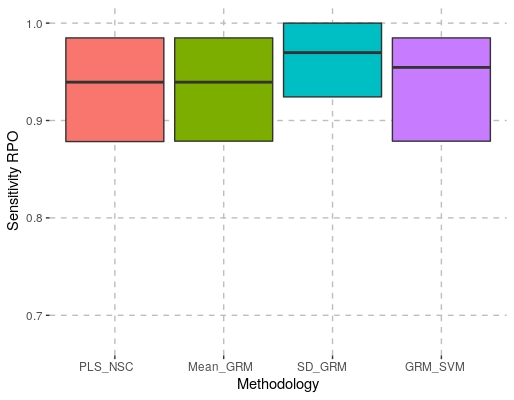


**Supplementary Figure S6.** Bootstrap confidence interval of 95% and results of the pairwise Student’s T-test for the sensitivity of RPO for RS1. Non significant differences are not represented.


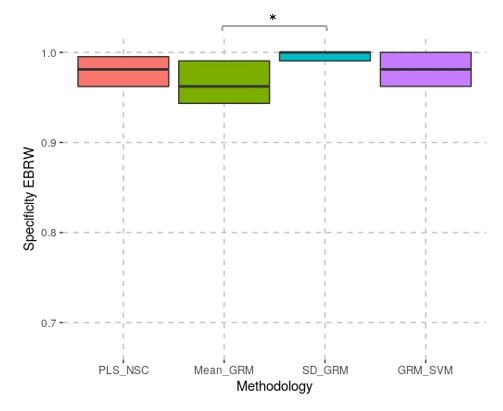


**Supplementary Figure S7.** Bootstrap confidence interval of 95% and results of the pairwise Student’s T-test for the specificity of EBRW for RS1. Non significant differences are not represented. *: significant difference.


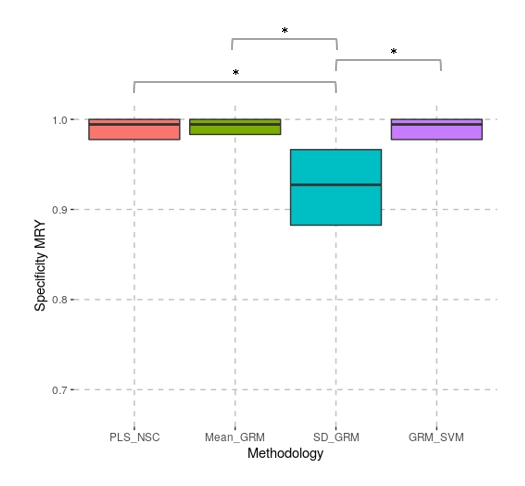


**Supplementary Figure S8.** Bootstrap confidence interval of 95% and results of the pairwise Student’s T-test for the specificity of MRY for RS1. Non significant differences are not represented. *: significant difference.


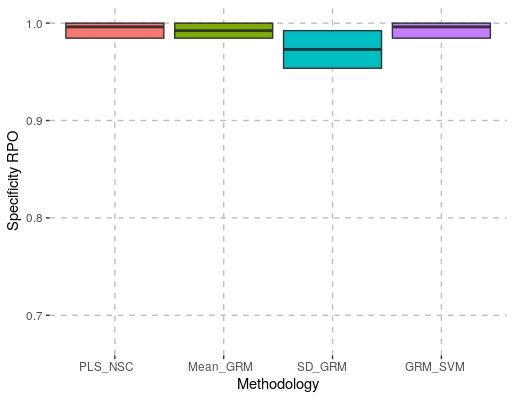


**Supplementary Figure S9.** Bootstrap confidence interval of 95% and results of the pairwise Student’s T-test for the specificity of RPO for RS1. Non significant differences are not represented. *: significant difference.


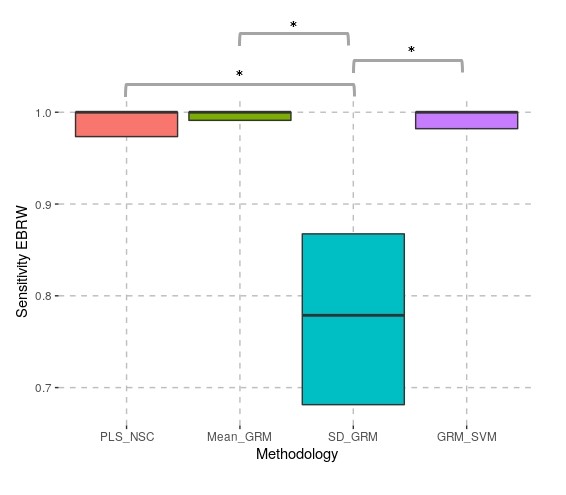
**Supplementary Figure S10.** Bootstrap confidence interval of 95% and results of the pairwise Student’s T-test for the sensitivity of EBRW for RS2. Non significant differences are not represented. *: significant difference.


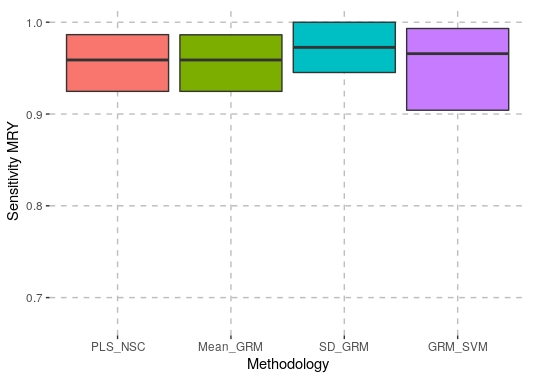


**Supplementary Figure S11.** Bootstrap confidence interval of 95% and results of the pairwise Student’s T-test for the sensitivity of MRY for RS2. Non significant differences are not represented.


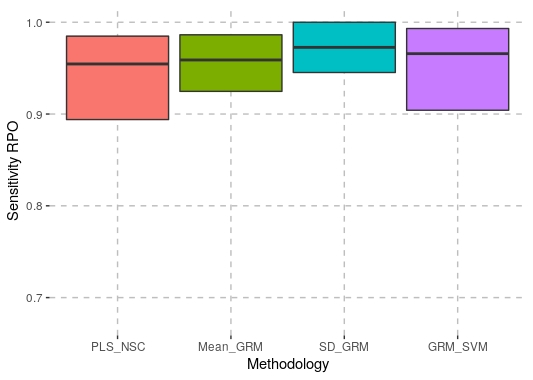


**Supplementary Figure S12.** Bootstrap confidence interval of 95% and results of the pairwise Student’s T-test for the sensitivity of RPO for RS2. Non significant differences are not represented.


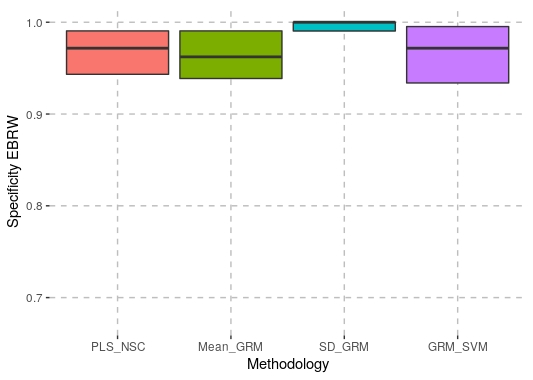


**Supplementary Figure S13.** Bootstrap confidence interval of 95% and results of the pairwise Student’s T-test for the specificity of EBRW for RS2. Non significant differences are not represented.


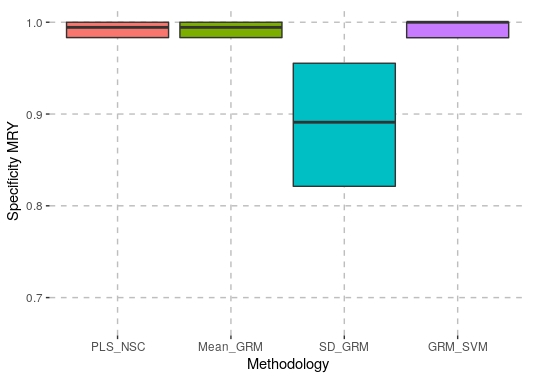


**Supplementary Figure S14.** Bootstrap confidence interval of 95% and results of the pairwise Student’s T-test for the specificity of MRY for RS2. Non significant differences are not represented.


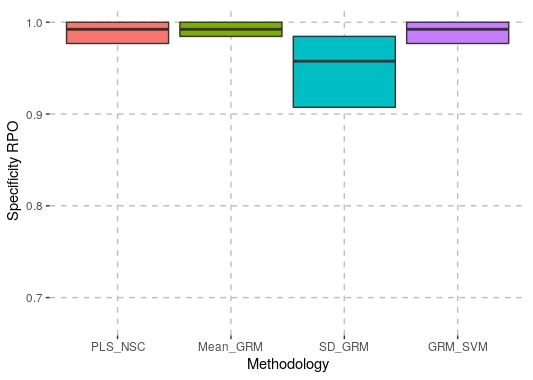


**Supplementary Figure S15.** Bootstrap confidence interval of 95% and results of the pairwise Student’s T-test for the specificity of RPO for RS2. Non significant differences are not represented.
