# Supplementary material for: The use of a genomic relationship matrix for breed assignment of cattle breeds: comparison and combination with a machine learning method
Source: J Anim Sci. 2023 May 23;101:skad172. doi: 10.1093/jas/skad172 (PMC10276639; doi:10.1093/jas/skad172)
Supplement: skad172_suppl_Supplementary_File [file skad172_suppl_supplementary_file.docx]

Supplementary File S1

Methodologies mean_GRM, SD_GRM and GRM_SVM

Hélène Wilmot et al

27 April 2023

The objective of this R script is to explain the development of the different methodologies developed. These methodologies are based on the genomic relationship matrix (GRM). Mean_GRM refers to the assignment based on the highest mean relatedness of an animal to the breeds found in the reference set. SD_GRM refers to the assignment based on the highest standard deviation (SD) of the relatedness of an animal to the breeds found in the reference set. GRM_SVM refers to the assignment based on a linear support vector machine (SVM) using the different values of mean and SD of the relatedness of the animal to be assigned as an input. The Supplemental material of Wilmot et al. (2022): <https://doi.org/10.1111/jbg.12643> is a Rscript to implement the PLS_NSC methodology.

Methodology applied for the validation animals can be used in routine for any new animal to be breed-assigned.

# Working directory should be set

# Used packages

#install.packages("dplyr",repos = "http://cran.us.r-project.org")
library(dplyr)

##
## Attaching package : 'dplyr'

## The following objects are masked from 'package:stats':
##
## filter, lag

## The following objects are masked from 'package:base':
##
## intersect, setdiff, setequal, union

#install.packages("caret",repos = "http://cran.us.r-project.org")
library(caret) #To cross-validate and validate GRM_SVM

## Loading required package : ggplot2

## Loading required package : lattice

#install.packages("matrixStats",repos = "http://cran.us.r-project.org")
library(matrixStats)

##
## Attaching package : 'matrixStats'

## The following object is masked from 'package:dplyr':
##
## count

# 1. Mean_GRM

## 1.1. Computation of mean relatedness of each validation animal to each breed of the reference set

#GRM computed with calc_grm software
GRM_valid<-read.table(file="G.grm", header=FALSE)
head(GRM_valid)

## V1 V2 V3 V4 V5
## 1 1 1 1.03713406 1 1
## 2 2 1 0.07455578 2 1
## 3 2 2 1.03317129 2 2
## 4 3 1 0.02709459 3 1
## 5 3 2 0.04037747 3 2
## 6 3 3 1.08186834 3 3

#V1 and V2 are number order assigned to animals , V3 is the relatedness, V4 and V5 are number IDs (here identical to V1 and V2)
GRM_valid<-subset(GRM_valid, V1>325 & V2<326) #Keep relatedness of validation animals to reference animals only

#Get the IDs of the reference animals
ID_RS1<-read.table(file=paste("1_RS1_ID.txt", sep=""), header=FALSE, sep="") #V1 is the breed, V2 is the Interbull ID and V3 is the number ID
ID_EBRW_RS1<-subset(ID_RS1, V1=="EBRW") #IDs of EBRW reference animals
ID_MRY_RS1<-subset(ID_RS1, V1=="MRY") #IDs of MRY reference animals
ID_RPO_RS1<-subset(ID_RS1, V1=="RPO") #IDs of RPO reference animals

GRM_valid_EBRW<-dplyr::filter(GRM_valid, V2 %in% ID_EBRW_RS1$V3) #Keep relatedness of validation animals to the EBRW reference set
mean_to_EBRW<- GRM_valid_EBRW %>% group_by(V1) %>%
 dplyr::summarise(mean_relatedness_EBRW=mean(V3)) #Compute the mean relatedness of validation animals to the EBRW reference set
head(mean_to_EBRW)

## # A tibble: 6 × 2
## V1 mean_relatedness_EBRW
## <int> <dbl>
## 1 326 0.0379
## 2 327 0.0319
## 3 328 0.0340
## 4 329 0.0442
## 5 330 0.0349
## 6 331 0.0518

GRM_valid_MRY<-dplyr::filter(GRM_valid, V2 %in% ID_MRY_RS1$V3) #Keep relatedness of validation animals to the MRY reference set
mean_to_MRY<- GRM_valid_MRY %>% group_by(V1) %>%
 dplyr::summarise(mean_relatedness_MRY=mean(V3)) #Compute the mean relatedness of validation animals to the MRY reference set

GRM_valid_RPO<- dplyr::filter(GRM_valid, V2 %in% ID_RPO_RS1$V3) #Keep relatedness of validation animals to the RPO reference set
mean_to_RPO<- GRM_valid_RPO %>% group_by(V1) %>%
 dplyr::summarise(mean_relatedness_RPO=mean(V3)) #Compute the mean relatedness of validation animals to the RPO reference set

## 1.2. Assignment of validation animals to their breed based on mean_GRM

#Merging mean relatedness of validation animals to each breed of interest
mergedrel<-merge(mean_to_EBRW, mean_to_MRY, by=intersect("V1","V1"))
mergedrel2<-merge(mergedrel, mean_to_RPO, by=intersect("V1","V1"))
#Giving the real breed of origin of validation animals
mergedrel2$Breed<-rep(c("EBRW","RPO","MRY"),c(113,66,146))
mergedrel2 <- transform(mergedrel2, predicted=c("EBRW", "MRY", "RPO")[max.col(mergedrel2[-c(1,5)])]) #Predict the breed of origin of validation animals based on the highest mean relatedness
#Determine if predicted breed is the same as the breed of origin
mergedrel2$diff<-ifelse(mergedrel2$Breed == mergedrel2$predicted, 1,0)
head(mergedrel2)

## V1 mean_relatedness_EBRW mean_relatedness_MRY mean_relatedness_RPO Breed
## 1 326 0.03792602 -0.02561825 -0.012307757 EBRW
## 2 327 0.03190195 -0.02494998 -0.006951994 EBRW
## 3 328 0.03398034 -0.01413209 -0.019848224 EBRW
## 4 329 0.04424611 -0.02970113 -0.014544990 EBRW
## 5 330 0.03487866 -0.02045412 -0.014424537 EBRW
## 6 331 0.05178547 -0.03669798 -0.015087471 EBRW
## predicted diff
## 1 EBRW 1
## 2 EBRW 1
## 3 EBRW 1
## 4 EBRW 1
## 5 EBRW 1
## 6 EBRW 1

percentage<-(sum(mergedrel2$diff)/nrow(mergedrel2)) #Percentage of correct assignment=global accuracy
percentage

## [1] 0.9723077

# 2. SD_GRM

## 2.1. Computation of SD of the relatedness of each validation animal to each breed of the reference set

#If performed alone, same steps as mean_GRM must be computed before computation of the SD of the relatedness of validation animals
sd_to_EBRW<- GRM_valid_EBRW %>% group_by(V1) %>%
dplyr::summarise(sd_relatedness_EBRW=sd(V3)) #Compute the SD of the relatedness of validation animals to the EBRW reference set
head(sd_to_EBRW)

## # A tibble: 6 × 2
## V1 sd_relatedness_EBRW
## <int> <dbl>
## 1 326 0.0606
## 2 327 0.0130
## 3 328 0.0376
## 4 329 0.0614
## 5 330 0.0635
## 6 331 0.0746

sd_to_MRY<- GRM_valid_MRY %>% group_by(V1) %>%
dplyr::summarise(sd_relatedness_MRY=sd(V3)) #Compute the SD of the relatedness of validation animals to the MRY reference set
sd_to_RPO<- GRM_valid_RPO %>% group_by(V1) %>%
dplyr::summarise(sd_relatedness_RPO=sd(V3)) #Compute the SD of the relatedness of validation animals to the RPO reference set

## 1.2. Assignment of validation animals to their breed based on SD_GRM

#Merging SD of the relatedness of validation animals to each breed of interest
mergedrelSD<-merge(sd_to_EBRW, sd_to_MRY, by=intersect("V1","V1"))
mergedrelSD2<-merge(mergedrelSD, sd_to_RPO, by=intersect("V1","V1"))
#Giving the real breed of origin of validation animals
mergedrelSD2$Breed<-rep(c("EBRW","RPO","MRY"),c(113,66,146))
mergedrelSD2 <- transform(mergedrelSD2, predicted=c("EBRW", "MRY", "RPO")[max.col(mergedrelSD2[-c(1,5)])])#Predict the breed of origin of validation animals based on the highest SD of the relatedness
#Determine if predicted breed is the same as the breed of origin
mergedrelSD2$diff<-ifelse(mergedrelSD2$Breed == mergedrelSD2$predicted, 1,0)
head(mergedrelSD2)

## V1 sd_relatedness_EBRW sd_relatedness_MRY sd_relatedness_RPO Breed predicted
## 1 326 0.06055806 0.01678730 0.02555644 EBRW EBRW
## 2 327 0.01300901 0.02035456 0.02022849 EBRW MRY
## 3 328 0.03762712 0.02063230 0.01535479 EBRW EBRW
## 4 329 0.06140827 0.01937241 0.01564975 EBRW EBRW
## 5 330 0.06349364 0.01698870 0.01567396 EBRW EBRW
## 6 331 0.07463982 0.01855827 0.01791603 EBRW EBRW
## diff
## 1 1
## 2 0
## 3 1
## 4 1
## 5 1
## 6 1

percentageSD<-(sum(mergedrelSD2$diff)/nrow(mergedrelSD2))#Percentage of correct assignment=global accuracy
percentageSD

## [1] 0.9353846

# 3. GRM_SVM

## 3.1. Formating

Transform the table obtained in calc_grm as a matrix, easier to handle for the GRM_SVM methodology

IN <- data.table::fread("G.grm") #Read the GRM table
# Column in V1
# Row in V2
n.entries <- max(IN$V5) #To determine the dimension of the GRM matrix
m <- matrix(9, nrow = n.entries, ncol = n.entries) #Set an empty matrix with the correct dimensions
#A loop to fill the matrix with the values of the GRM table
counter <- 1
for(col.i in 1:ncol(m)){
for(row.j in 1:col.i){
m[row.j, col.i] <- IN$V3[counter]
m[col.i, row.j] <- IN$V3[counter]
counter <- counter + 1
}
}
m<-m[1:325,1:325] #Keep reference animals
colnames(m)<-c(1:325) #Assign the ID of animals as column names
diag(m)<-NA #Replace self-relatedness (on the diagonal) by NA to avoid a bias in the computation of mean relatedness
m[1:5,1:5]

## 1 2 3 4 5
## [1,] NA 0.07455578 0.02709459 0.02822828 0.04904310
## [2,] 0.07455578 NA 0.04037747 0.05309095 0.03405326
## [3,] 0.02709459 0.04037747 NA 0.03608309 0.14149051
## [4,] 0.02822828 0.05309095 0.03608309 NA 0.01336672
## [5,] 0.04904310 0.03405326 0.14149051 0.01336672 NA

## 3.2. Computation of mean and SD of the relatedness within the reference set (self-relatedness excluded)

EBRW_list<-ID_EBRW_RS1$V3 #List of EBRW reference animals
MRY_list<-ID_MRY_RS1$V3 #List of MRY reference animals
RPO_list<-ID_RPO_RS1$V3 #List of RPO reference animals
#Mean relatedness to EBRW animals within RS1
forComSVMEBRW<-as.data.frame(rowMeans(subset(m,select=EBRW_list),na.rm=TRUE))
forComSVMEBRW$ID<-rownames(forComSVMEBRW)#Set IDs in the file
head(forComSVMEBRW)

## rowMeans(subset(m, select = EBRW_list), na.rm = TRUE) ID
## 1 0.04835851 1
## 2 0.04855461 2
## 3 0.04950669 3
## 4 0.03132637 4
## 5 0.04080591 5
## 6 0.03051656 6

#Mean relatedness to MRY animals within RS1
forComSVMMRY<-as.data.frame(rowMeans(subset(m,select=MRY_list),na.rm=TRUE))
forComSVMMRY$ID<-rownames(forComSVMMRY) #Set IDs in the file

#Mean relatedness to RPO animals within RS1
forComSVMRPO<-as.data.frame(rowMeans(subset(m,select=RPO_list),na.rm=TRUE))
forComSVMRPO$ID<-rownames(forComSVMRPO)#Set IDs in the file

#SD of the relatedness to EBRW animals within RS1
forComSVMEBRWsd<-as.data.frame(rowSds(subset(m,select=EBRW_list),na.rm=TRUE))
forComSVMEBRWsd$ID<-rownames(forComSVMEBRWsd)#Set IDs in the file
head(forComSVMEBRWsd)

## rowSds(subset(m, select = EBRW_list), na.rm = TRUE) ID
## 1 0.07057492 1
## 2 0.08162876 2
## 3 0.06592701 3
## 4 0.03301942 4
## 5 0.02547497 5
## 6 0.05181382 6

#SD of the relatedness to MRY animals within RS1
forComSVMMRYsd<-as.data.frame(rowSds(subset(m,select=MRY_list),na.rm=TRUE))
forComSVMMRYsd$ID<-rownames(forComSVMMRYsd)#Set IDs in the file

#SD of the relatedness to RPO animals within RS1
forComSVMRPOsd<-as.data.frame(rowSds(subset(m,select=RPO_list),na.rm=TRUE))
forComSVMRPOsd$ID<-rownames(forComSVMRPOsd)#Set IDs in the file

#Merge the six variables (mean and SD of the relatedness within RS1)
combi1 <- merge(forComSVMEBRW, forComSVMMRY, by=intersect("ID","ID"))
combi2<-merge(forComSVMRPO,combi1, by=intersect("ID","ID"))
combi3<-merge(forComSVMEBRWsd,combi2, by=intersect("ID","ID"))
combi4<-merge(forComSVMMRYsd,combi3, by=intersect("ID","ID"))
combi5<-merge(forComSVMRPOsd,combi4, by=intersect("ID","ID"))
#Define column names
colnames(combi5)<-c("ID","sd_RPO", "sd_MRY","sd_EBRW","relatedness_RPO","relatedness_EBRW","relatedness_MRY")
combi5$ID<-as.numeric(combi5$ID) #Define ID as numeric
combi5<-combi5[order(combi5$ID),] #Order by ID
combi5$Breed<-ID_RS1$V1 #Define breed of origin
combi_final<-combi5[,c(1,6,7,5,4,3,2,8)] #Order columns (important to have the same order for validation)
combi_final$Breed<-as.factor(combi_final$Breed) #Define breed as a factor
levels(combi_final$Breed) #Check the factor levels of the breed

## [1] "EBRW" "MRY" "RPO"

head(combi_final)

## ID relatedness_EBRW relatedness_MRY relatedness_RPO sd_EBRW sd_MRY
## 1 1 0.04835851 -0.03382193 -0.02328679 0.07057492 0.01706299
## 112 2 0.04855461 -0.03615713 -0.02111089 0.08162876 0.02019785
## 223 3 0.04950669 -0.03439136 -0.02425126 0.06592701 0.02039905
## 260 4 0.03132637 -0.02753421 -0.01312367 0.03301942 0.01780199
## 271 5 0.04080591 -0.03329305 -0.01663848 0.02547497 0.01986704
## 282 6 0.03051656 -0.02163934 -0.01780549 0.05181382 0.01882033
## sd_RPO Breed
## 1 0.01604079 EBRW
## 112 0.01545846 EBRW
## 223 0.01886361 EBRW
## 260 0.01568492 EBRW
## 271 0.01863172 EBRW
## 282 0.01302048 EBRW

## 3.3. Training of the linear SVM on the values of the reference set

set.seed(1001) # To fix the algorithm
ctrl<-trainControl(method="repeatedcv", number=10, classProbs=TRUE, selectionFunction= "best") # Parameter optimized based on the best value obtained in 10 fold cross-validation

grid<-expand.grid(C=c(0.001,0.01,0.1,0.2,0.3,0.4,0.5, 0.6, 0.7, 0.8, 0.9)) #Cost values to be tested
row.names(combi_final)<-combi_final$ID #Set ID in rownames
combi_final_svm<-combi_final[,-1] #Remove the variable ID
svmL = train(Breed~.,data=combi_final_svm,
 method="svmLinear",
 trControl = ctrl,
 preProc=c("center","scale"), #Mean centring and SD scaling for each of the six variables
 metric="Accuracy",
 tuneGrid=grid)
svmL

## Support Vector Machines with Linear Kernel
##
## 325 samples
## 6 predictor
## 3 classes: 'EBRW', 'MRY', 'RPO'
##
## Pre-processing: centered (6), scaled (6)
## Resampling: Cross-Validated (10 fold, repeated 1 times)
## Summary of sample sizes: 294, 292, 291, 293, 292, 293, ...
## Resampling results across tuning parameters:
##
## C Accuracy Kappa
## 0.001 0.9535868 0.9279109
## 0.010 0.9721474 0.9566731
## 0.100 0.9662651 0.9471640
## 0.200 0.9722365 0.9568005
## 0.300 0.9722365 0.9568005
## 0.400 0.9753615 0.9617464
## 0.500 0.9722365 0.9568005
## 0.600 0.9753615 0.9617464
## 0.700 0.9724204 0.9569204
## 0.800 0.9724204 0.9569204
## 0.900 0.9724204 0.9569204
##
## Accuracy was used to select the optimal model using the largest value.
## The final value used for the model was C = 0.4.

## 3.4. Validation of the linear SVM on the validation set

#Merge files with the mean and SD of the relatedness of the validation set
#These were previously computed on sections 1.1. and 2.1.
combination<-merge(mergedrel2, mergedrelSD2, by=intersect("V1","V1"))
combination<-combination[,c(1:4,8:11)] #Same order of columns as for the input file used for training
colnames(combination)<-c("ID","relatedness_EBRW","relatedness_MRY","relatedness_RPO","sd_EBRW","sd_MRY","sd_RPO","Breed") #Define column names as previously
combination$Breed<-as.factor(combination$Breed) #Define breed as a factor
combination_svm<-combination[,-1] #Remove the variable ID
levels(combination_svm$Breed) #Check the factor levels of the breed

## [1] "EBRW" "MRY" "RPO"

head(combination_svm)

## relatedness_EBRW relatedness_MRY relatedness_RPO sd_EBRW sd_MRY
## 1 0.03792602 -0.02561825 -0.012307757 0.06055806 0.01678730
## 2 0.03190195 -0.02494998 -0.006951994 0.01300901 0.02035456
## 3 0.03398034 -0.01413209 -0.019848224 0.03762712 0.02063230
## 4 0.04424611 -0.02970113 -0.014544990 0.06140827 0.01937241
## 5 0.03487866 -0.02045412 -0.014424537 0.06349364 0.01698870
## 6 0.05178547 -0.03669798 -0.015087471 0.07463982 0.01855827
## sd_RPO Breed
## 1 0.02555644 EBRW
## 2 0.02022849 EBRW
## 3 0.01535479 EBRW
## 4 0.01564975 EBRW
## 5 0.01567396 EBRW
## 6 0.01791603 EBRW

pred<-predict(svmL, combination_svm) #Predict the breed based on the model tuned by cross-validation (section 3.3.)
confus<-confusionMatrix(pred, combination_svm[,7])
confus #Confusion matrix with global accuracy, sensitivities and specificities for each breed

## Confusion Matrix and Statistics
##
## Reference
## Prediction EBRW MRY RPO
## EBRW 112 0 1
## MRY 0 144 0
## RPO 1 2 65
##
## Overall Statistics
##
## Accuracy : 0.9877
## 95% CI : (0.9688, 0.9966)
## No Information Rate : 0.4492
## P-Value [Acc > NIR] : < 2.2e-16
##
## Kappa : 0.9807
##
## Mcnemar's Test P-Value : NA
##
## Statistics by Class:
##
## Class: EBRW Class: MRY Class: RPO
## Sensitivity 0.9912 0.9863 0.9848
## Specificity 0.9953 1.0000 0.9884
## Pos Pred Value 0.9912 1.0000 0.9559
## Neg Pred Value 0.9953 0.9890 0.9961
## Prevalence 0.3477 0.4492 0.2031
## Detection Rate 0.3446 0.4431 0.2000
## Detection Prevalence 0.3477 0.4431 0.2092
## Balanced Accuracy 0.9932 0.9932 0.9866
