# Supplementary material for: The use of a genomic relationship matrix for breed assignment of cattle breeds: comparison and combination with a machine learning method
Source: J Anim Sci. 2023 May 23;101:skad172. doi: 10.1093/jas/skad172 (PMC10276639; doi:10.1093/jas/skad172)
Supplement: skad172_suppl_Supplementary_Table_S3 [file skad172_suppl_supplementary_table_s3.docx]

**Supplementary Table S3.**

For animals in RS1, within- and between-breed correlations between the mean relatedness and the SD of the relatedness averaged across 200 repetitions.

| **Breed 1** | **Breed 2** | **Mean correlation between mean relatedness and the SD of the relatedness** | **SD of the correlation between mean relatedness and the SD of the relatedness** |
| --- | --- | --- | --- |
| EBRW | EBRW | 0.65 | 0.050 |
|  | MRY | -0.13 | 0.099 |
|  | RPO | 0.40 | 0.155 |
| MRY | EBRW | -0.18 | 0.120 |
|  | MRY | 0.55 | 0.047 |
|  | RPO | 0.31 | 0.147 |
| RPO | EBRW | 0.47 | 0.120 |
|  | MRY | 0.51 | 0.091 |
|  | RPO | 0.79 | 0.041 |
