# Supplementary material for: The use of a genomic relationship matrix for breed assignment of cattle breeds: comparison and combination with a machine learning method
Source: J Anim Sci. 2023 May 23;101:skad172. doi: 10.1093/jas/skad172 (PMC10276639; doi:10.1093/jas/skad172)
Supplement: skad172_suppl_Supplementary_Table_S5-S10 [file skad172_suppl_supplementary_table_s5-s10.docx]

**Supplementary Table S5.**

Minimum, mean, maximum and SD of the sensitivity for the EBRW breed for each combination of methodology and reference set, across 200 repetitions.

| **Reference set** | **Methodology^1^** | | | |
| --- | --- | --- | --- | --- |
|  | **PLS_NSC** | **Mean_GRM** | **SD_GRM** | **GRM_SVM** |
| RS1 |  |  |  |  |
| Minimum sensitivity, % | 98.23 | 100 | 77.88 | 98.23 |
| Mean sensitivity, % | 99.94^a^ | 100^a^ | 86.75^b^ | 99.93^a^ |
| Maximum sensitivity, % | 100 | 100 | 94.69 | 100 |
| SD of the sensitivity, % | 0.243 | 0 | 3.473 | 0.307 |
| RS2 |  |  |  |  |
| Minimum sensitivity, % | 96.46 | 98.23 | 64.60 | 93.81 |
| Mean sensitivity, % | 99.52^a^ | 99.92^a^ | 77.96^b^ | 99.74^a^ |
| Maximum sensitivity, % | 100 | 100 | 91.15 | 100 |
| SD of the sensitivity, % | 0.720 | 0.302 | 4.889 | 0.725 |

^1^: Methodologies with the same letter have not significantly different mean sensitivities within a reference set (*P* < 0.0083 with the Bonferroni correction).

**Supplementary Table S6.**

Minimum, mean, maximum and SD of the specificity for the EBRW breed for each combination of methodology and reference set, across 200 repetitions.

| **Reference set** | **Methodology^1^** | | | |
| --- | --- | --- | --- | --- |
|  | **PLS_NSC** | **Mean_GRM** | **SD_GRM** | **GRM_SVM** |
| RS1 |  |  |  |  |
| Minimum specificity, % | 94.81 | 93.40 | 98.58 | 95.75 |
| Mean specificity, % | 97.95^a,b^ | 96.36^b^ | 99.89^a^ | 98.27^a,b^ |
| Maximum specificity, % | 100 | 99.06 | 100 | 100 |
| SD of the specificity, % | 0.970 | 1.215 | 0.247 | 0.899 |
| RS2 |  |  |  |  |
| Minimum specificity, % | 91.98 | 92.92 | 98.11 | 90.57 |
| Mean specificity, % | 96.96^a^ | 96.29^a^ | 99.77^a^ | 96.98^a^ |
| Maximum specificity, % | 99.53 | 99.06 | 100 | 100 |
| SD of the specificity, % | 1.267 | 1.263 | 0.354 | 1.624 |

^1^: Methodologies with the same letter have not significantly different mean specificities within a reference set (*P* < 0.0083 with the Bonferroni correction).

**Supplementary Table S7.**

Minimum, mean, maximum and SD of the sensitivity for the MRY breed for each combination of methodology and reference set, across 200 repetitions.

| **Reference set** | **Methodology^1^** | | | |
| --- | --- | --- | --- | --- |
|  | **PLS_NSC** | **Mean_GRM** | **SD_GRM** | **GRM_SVM** |
| RS1 |  |  |  |  |
| Minimum sensitivity, % | 94.52 | 92.47 | 95.21 | 93.15 |
| Mean sensitivity, % | 98.30^a^ | 95.86^a^ | 97.84^a^ | 97.99^a^ |
| Maximum sensitivity, % | 100 | 99.32 | 100 | 100 |
| SD of the sensitivity, % | 1.137 | 1.392 | 1.102 | 1.346 |
| RS2 |  |  |  |  |
| Minimum sensitivity, % | 90.41 | 91.10 | 93.15 | 87.67 |
| Mean sensitivity, % | 95.99^a^ | 95.61^a^ | 97.41^a^ | 96.01^a^ |
| Maximum sensitivity, % | 99.32 | 99.32 | 100 | 100 |
| SD of the sensitivity, % | 1.772 | 1.537 | 1.285 | 2.194 |

^1^: Methodologies with the same letter have not significantly different mean sensitivities within a reference set (*P* < 0.0083 with the Bonferroni correction).

**Supplementary Table S8.**

Minimum, mean, maximum and SD of the specificity for the MRY breed for each combination of methodology and reference set, across 200 repetitions.

| **Reference set** | **Methodology^1^** | | | |
| --- | --- | --- | --- | --- |
|  | **PLS_NSC** | **Mean_GRM** | **SD_GRM** | **GRM_SVM** |
| RS1 |  |  |  |  |
| Minimum specificity, % | 97.77 | 97.21 | 87.15 | 95.53 |
| Mean specificity, % | 99.20^a^ | 99.58^a^ | 92.73^b^ | 99.22^a^ |
| Maximum specificity, % | 100 | 100 | 97.21 | 100 |
| SD of the specificity, % | 0.645 | 0.469 | 2.132 | 0.756 |
| RS2 |  |  |  |  |
| Minimum specificity, % | 97.77 | 97.77 | 78.77 | 97.77 |
| Mean specificity, % | 99.61^a^ | 99.55^a^ | 89.10^a^ | 99.57^a^ |
| Maximum specificity, % | 100 | 100 | 96.09 | 100 |
| SD of the specificity, % | 0.462 | 0.505 | 3.545 | 0.595 |

^1^: Methodologies with the same letter have not significantly different mean specificities within a reference set (*P* < 0.0083 with the Bonferroni correction).

**Supplementary Table S9.**

Minimum, mean, maximum and SD of the sensitivity for the RPO breed for each combination of methodology and reference set, across 200 repetitions.

| **Reference set** | **Methodology^1^** | | | |
| --- | --- | --- | --- | --- |
|  | **PLS_NSC** | **Mean_GRM** | **SD_GRM** | **GRM_SVM** |
| RS1 |  |  |  |  |
| Minimum sensitivity, % | 81.82 | 86.36 | 90.91 | 84.85 |
| Mean sensitivity, % | 93.33^a^ | 93.87^a^ | 97.05^a^ | 94.55^a^ |
| Maximum sensitivity, % | 100 | 98.48 | 100 | 100 |
| SD of the sensitivity, % | 2.988 | 2.557 | 2.114 | 2.850 |
| RS2 |  |  |  |  |
| Minimum sensitivity, % | 87.88 | 84.85 | 86.36 | 77.27 |
| Mean sensitivity, % | 95.15^a^ | 94.04^a^ | 96.11^a^ | 94.80^a^ |
| Maximum sensitivity, % | 100 | 98.48 | 100 | 100 |
| SD of the sensitivity, % | 2.387 | 2.560 | 2.245 | 3.213 |

^1^: Methodologies with the same letter have not significantly different mean sensitivities within a reference set (*P* < 0.0083 with the Bonferroni correction).

**Supplementary Table S10.**

Minimum, mean, maximum and SD of the specificity for the RPO breed for each combination of methodology and reference set, across 200 repetitions.

| **Reference set** | **Methodology^1^** | | | |
| --- | --- | --- | --- | --- |
|  | **PLS_NSC** | **Mean_GRM** | **SD_GRM** | **GRM_SVM** |
| RS1 |  |  |  |  |
| Minimum specificity, % | 98.46 | 98.46 | 93.05 | 97.68 |
| Mean specificity, % | 99.55^a^ | 99.37^a^ | 97.36^a^ | 99.40^a^ |
| Maximum specificity, % | 100 | 100 | 99.61 | 100 |
| SD of the specificity, % | 0.499 | 0.427 | 1.090 | 0.562 |
| RS2 |  |  |  |  |
| Minimum specificity, % | 96.91 | 98.07 | 88.80 | 95.37 |
| Mean specificity, % | 99.05^a^ | 99.32^a^ | 95.65^a^ | 99.08^a^ |
| Maximum specificity, % | 100 | 100 | 99.23 | 100 |
| SD of the specificity, % | 0.578 | 0.437 | 1.903 | 0.652 |

^1^: Methodologies with the same letter have not significantly different mean specificities within a reference set (*P* < 0.0083 with the Bonferroni correction).
